# Cardiac sarcoidosis presenting as multiple right intra-atrial masses mimicking cardiac tumor

**DOI:** 10.1186/s40959-024-00251-z

**Published:** 2024-08-08

**Authors:** Jay Gohri, Harshvardhan Luthra, Khushboo Qureshi, Karnati Prudhveer Reddy

**Affiliations:** 1 Department of Medicine, JSS Medical College and Hospital, Mysore, Karnataka India; 2 Department of Cardiology, Vinnitsa National Medical University named after M.I.Pirogov, Danyla Hoholytska 58a, Vinnytsia Oblast, 21036 Ukraine; 3 Department of Radiology, Vinnitsa National Medical University named after M.I.Pirogov, Vinnytsia, Ukraine; 4 Department of Cardiology, JSS Medical College and Hospital, Mysore, Karnataka India

**Keywords:** Cardiac sarcoidosis, Intra-cardiac mass, Cardiac imaging

## Abstract

**Background:**

Cardiac sarcoidosis though in itself, a rare entity, very rarely presents primarily with conduction abnormalities as the primary manifestation in the spectrum of presentations accounted by this chronic granulomatous systemic disease. Sarcoidosis presenting as intra-atrial masses is virtually unheard of.

**Case:**

A middle aged female presented with progressive conduction system disease was found to have right atrial masses of unclear etiologic on relevant imaging. Over the course of 3 months she underwent a dual-chamber ICD implant for her eventual complete heart block and a surgical resection following an inconclusive biopsy of the right atrial free wall mass. She was then diagnosed with cardiac sarcoidosis and started on immunosupressants almost instantaneously as a part of her treatment.

**Conclusion:**

This is an entirely new and unreported presentation of cardiac sarcoidosis as an intra-atrial mass. Through this case we bring light to cardiac sarcoidosis as a potential differential for intra-cardiac masses and how with available data do we go about treating it.

## Background

Sarcoidosis is a chronic multi-system granulomatous disease that may commonly be revealed as a diagnosis of exclusion when compared to other common granulomatous diseases, as its pathogenesis is not well understood [1]. General clinical manifestations of sarcoidosis have protean presentations, such as fever of unknown origin, weight loss, and lymphadenopathy, followed by more organ-specific symptoms like erythema nodosum, uveitis, uveo-parotid fever, and rarely neurological and renal involvement. The pathological finding revolves around the presence of non-caseating granulomas [2].

Cardiac sarcoidosis, being more common in women, has been found in almost 10% (with occult involvement estimated at 20–25%) of patients with systemic sarcoidosis, with a larger portion of patients remaining asymptomatic [3]. Cardiac involvement can occur at any time during the natural progression of sarcoidosis, sometimes being the only manifestation, with the prognosis solely determined by the extent of involvement. 18 F-FDG PET scan is now the investigation of choice, even in the presence of an inconclusive endomyocardial biopsy, and other modalities like cardiac MRI complement the diagnosis of cardiac sarcoidosis (CS). Cardiac involvement in sarcoidosis automatically escalates the severity of the disease, invariably requiring corticosteroid therapy with added immunosuppression provided by certain biologics [4]. The extremely rare presentation of sarcoidosis as an intra-cardiac mass adds to the diagnostic dilemma and the relatively unheard-of presentation of this condition.

### Case presentation

A female in her 40s presented to the emergency department with complaints of palpitations, dyspnea, and light-headedness. She had been experiencing intermittent palpitations and perceived skipped heartbeats for the preceding month, exacerbated by minimal exertion and bending over, causing discomfort. Dyspnea occurred solely during episodes of palpitations or upon exertion, with a solitary episode of light-headedness. The patient denied chest pain, syncope, or presyncope.

The patient’s family history was significant for pulmonary sarcoidosis in her father and systemic lupus erythematosus in two siblings. She did not consume alcohol or tobacco products. On examination, her general appearance was unremarkable, with a heart rate of 54 beats per minute, blood pressure of 136/88 mmHg, and no evidence of volume overload. Auscultation revealed distant consecutive cardiac sounds, with no other significant findings. Laboratory studies, including thyroid function tests, cardiac enzymes, comprehensive metabolic panel, and complete blood count, were within normal limits. The patient did not exhibit visual disturbances.

**Fig. 1:**
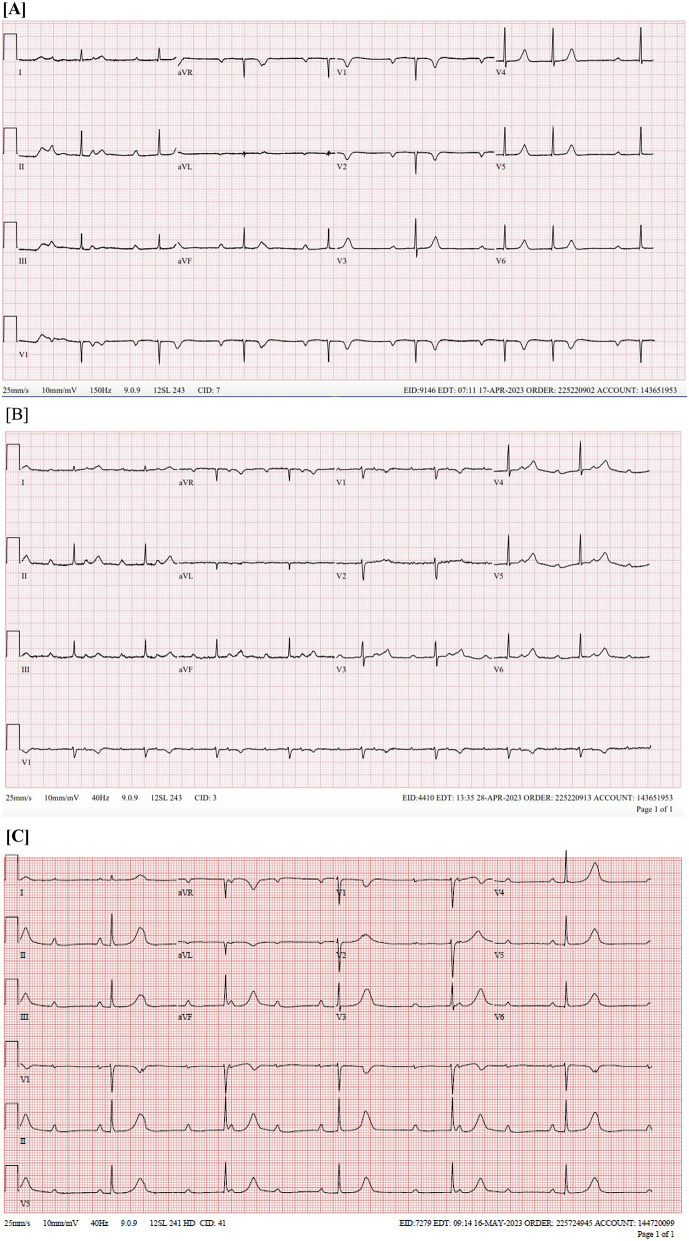
ECG and associated conduction disease progression(A to C). ECG shows very long (~ 500ms) first and intermittent Mobitz I second-degree AV block on initial presentation which eventually progresses onto complete heart block with severe bradycardia during the course in the hospital of the patient

Chest radiography showed no evidence of an acute cardiopulmonary process. The initial electrocardiogram demonstrated sinus bradycardia with prominent first-degree atrioventricular (AV) block, intermittent second-degree (Mobitz type 1, 2:1 AV block), and premature supraventricular complexes. Telemetry on the following day revealed second-degree type 2 AV block (most prominent) with the lowest heart rate in the 30–40 s range (Fig. 1).

Further cardiac workup revealed a normal echo with a 65% LVEF and a persistent 2:1 AV block on exercise stress testing.

**Fig. 2:**
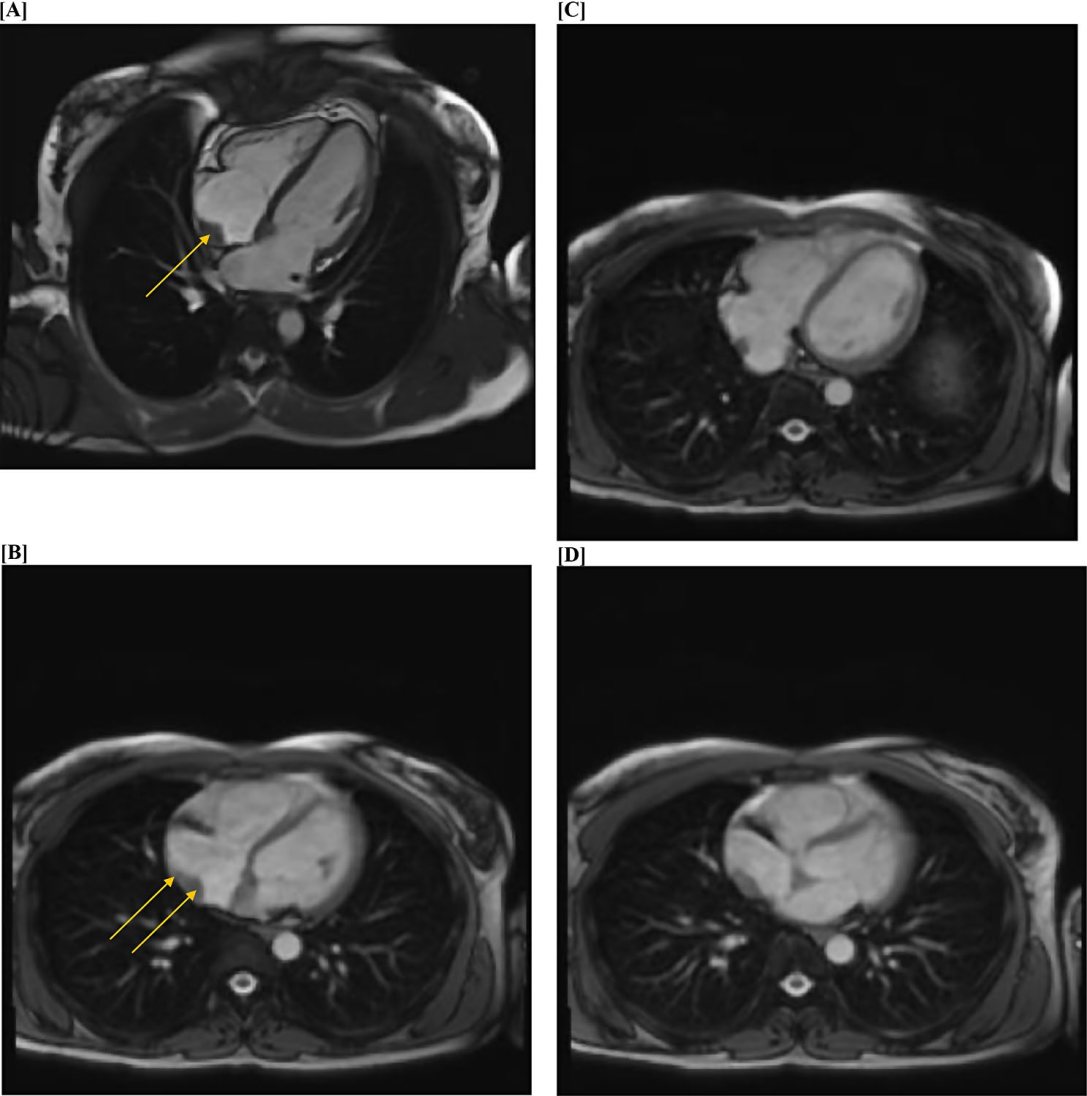
Cardiac MRI (**A**-**D**)

Cardiac magnetic resonance imaging (MRI) demonstrated the presence of two distinct masses within the right atrium: one measuring 27 × 14 mm along the posterior right atrial wall just superior to the inferior vena cava junction, and another more anterior mass measuring 18 × 19 mm located inferiorly along the interatrial septum. The masses were isointense on T1 and T2 sequences and did not exhibit signal suppression on fat-saturated imaging. Homogeneous perfusion was noted within the masses, along with delayed gadolinium enhancement consistent with vascularity and necrosis (initially raising suspicion for malignancy, which was subsequently ruled out by a negative whole-body computed tomography scan) (Fig. 2). Patient was then put on a cardiac diet before being scheduled for PET-CT.

**Fig. 3:**
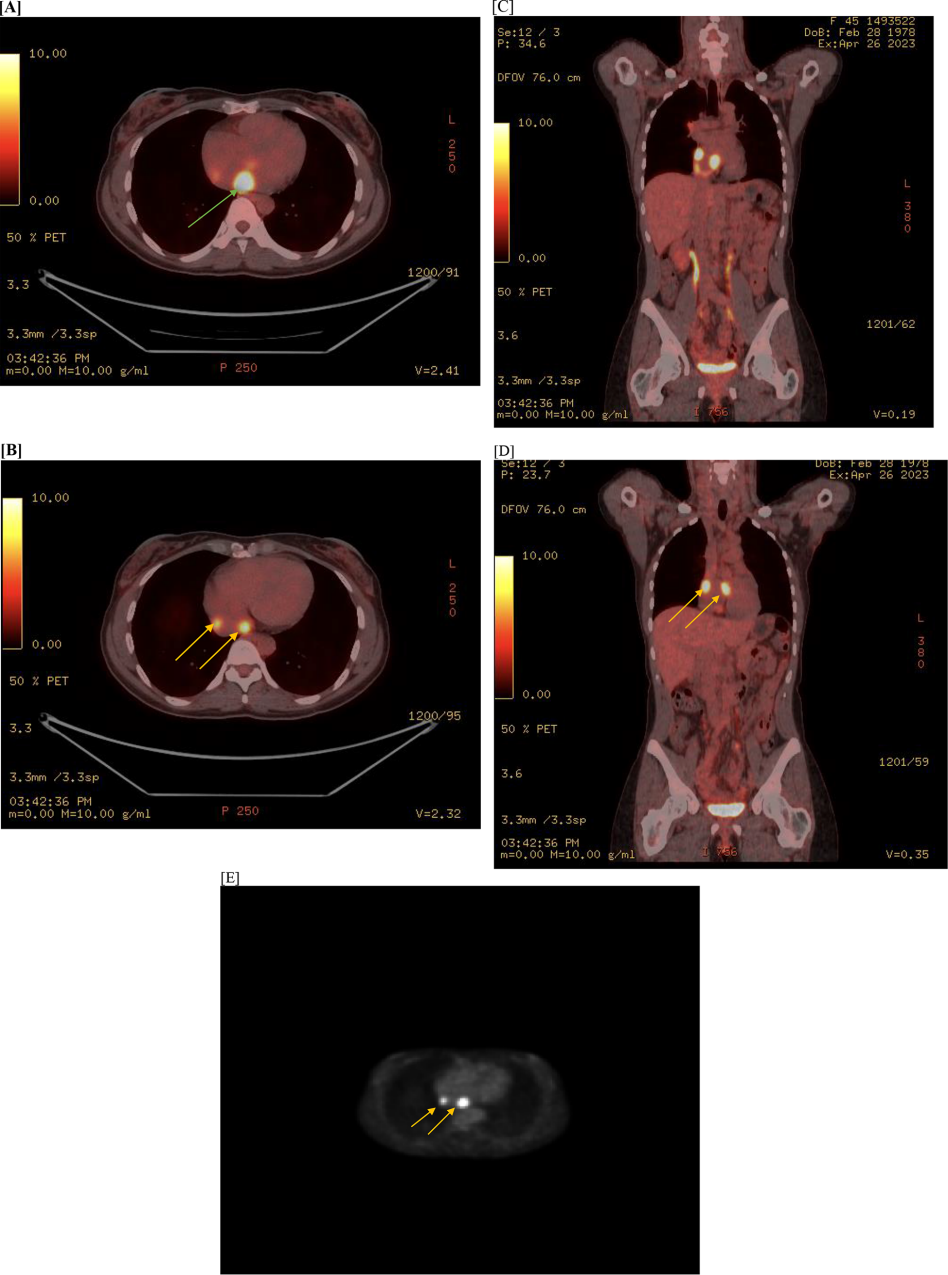
PET-CT(**A**-**E**)

18-FDG PET-CT findings revealed **s**everal foci of abnormal metabolism in the heart in the background of patchy activity; including at the level of the atrio-caval junction, anterior to the descending thoracic aorta and caudal to the left mainstem bronchus. Additional focus in the right atrium and an additional focus in the space between the 2 atria were also found with no evidence of pleural or pericardial effusion. A more subtle focus of uptake was also noted in the left ventricular apex. The most prominent foci(2 in number) were found in the right atrium(SUV- 8.14 and 8.48 respectively) with associated low level uptake within the bilateral hilar lymph node stations(FDG avid hilar nodes)(Fig. 3).

Repeat ECG showed complete heart block with narrow escape at 33 bpm. Patient was showing progression of conduction disease for which she ended up getting a temporary pacemaker.

Bronchoscopy done in view of avid hilar LN FDG uptake was inconclusive and endobronchial lung biopsy also showed no granulomas or malignancy.

**Fig. 4:**
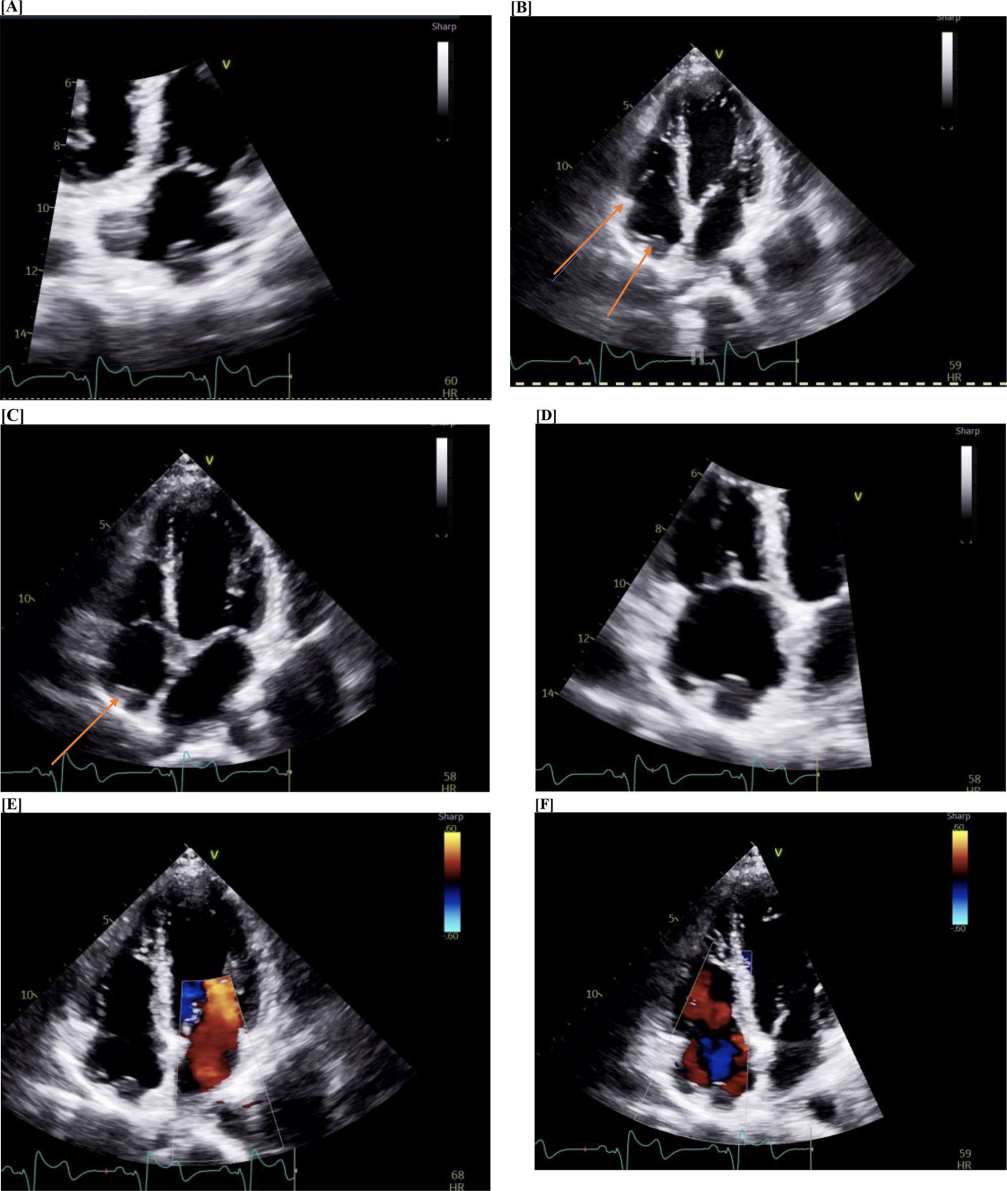
Transthoracic Echo Detecting Mass (A to F)

Limited Transesophageal echo done once again, a few months later, showed that there were 2 discrete, well-circumscribed, echogenic structures in the right atrium. The first structure measured 1.3 × 1.2 cm and was located in the superior aspect of the right atrium. The second structure measured 1.2 × 1.4 cm and was located in the inferior interatrial septal region (Fig. 4).

Patient underwent two endomyocardial biopsies, of which one was normal and the other was ICE guided. Intracardiac echocardiography (ICE) demonstrated a large, pedunculated mass arising from the membranous interventricular septum, extending superiorly and posteriorly into the interatrial septum, and then emerging in a posterior direction from the interatrial septum, covering the coronary sinus and extending to the posterior floor of the right atrium, posterior to the cavo-tricuspid isthmus. A second mass arose from the interatrial septum more superiorly, toward the superior vena cava-right atrial junction, but this was not as well-visualized on ICE compared to other imaging modalities. The latter showed presence of atypical spindle cells but was negative for cell markers, still making it difficult to rule out the possibility of angiosarcoma, hence requiring surgical removal of the mass in order to determine the basic pathology.

**Fig. 5:**
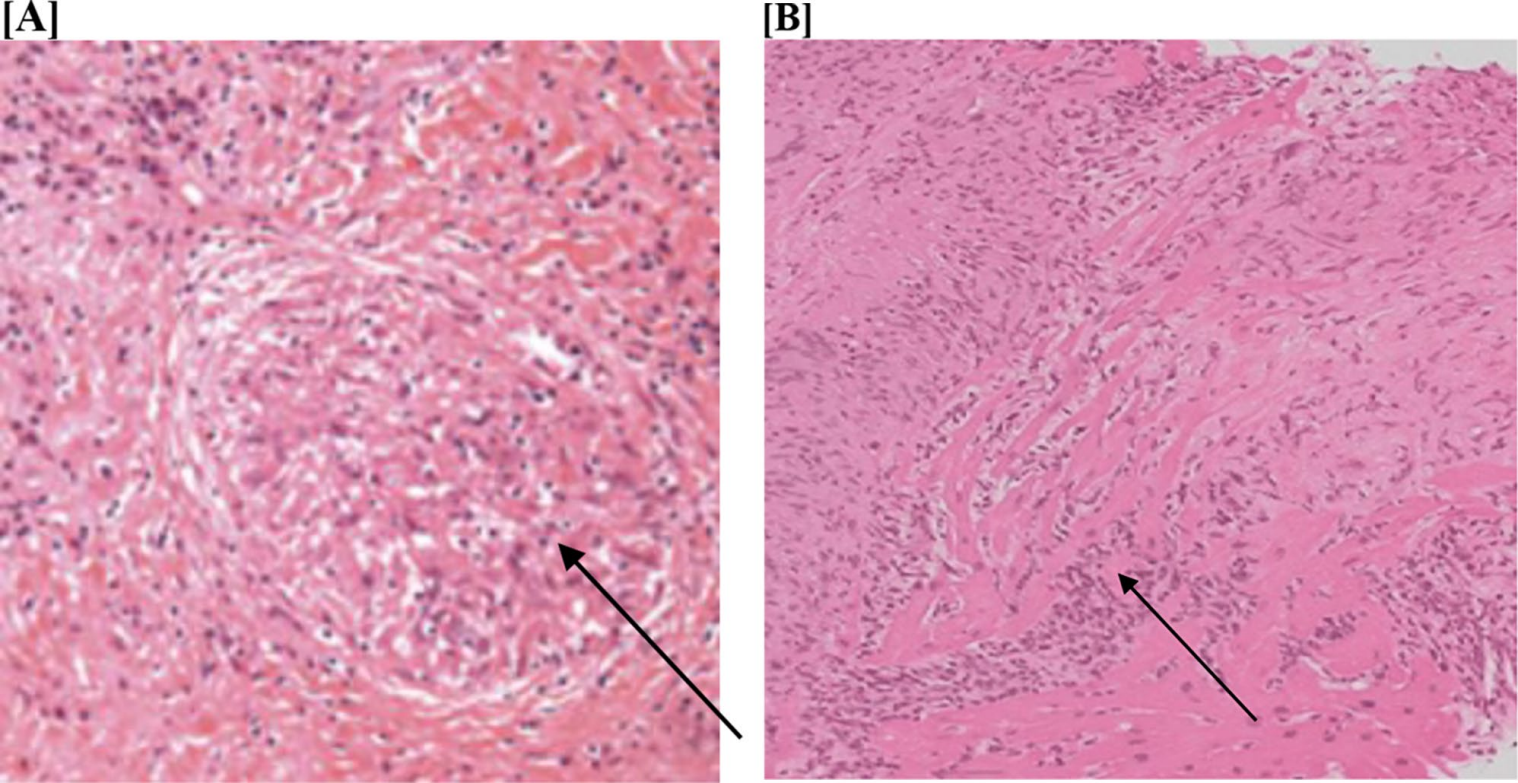
Histopathological report of the mass (**A**-**B**)

Intraoperative pathology demonstrated granulomas on frozen section, and the final surgical pathology revealed nodular, extensive myocardial involvement by non-necrotizing granulomatous inflammation, suggestive of sarcoid-type granulomas. Microscopic examination revealed numerous non-caseating granulomas, diffuse fibrosis, asteroid bodies, and giant cells, all consistent with sarcoidosis. These findings were not compatible with lymphoma, angiosarcoma, rheumatoid nodule, giant cell myocarditis, or a foreign body reaction (Fig. 5). Concurrently Patient’s blood sample was sent to check for levels of Angiotensin converting enzyme(ACE) which was increased(28.4) and there was increase in levels of soluble IL-12 receptor to 5844pg/ml.

**Fig. 6:**
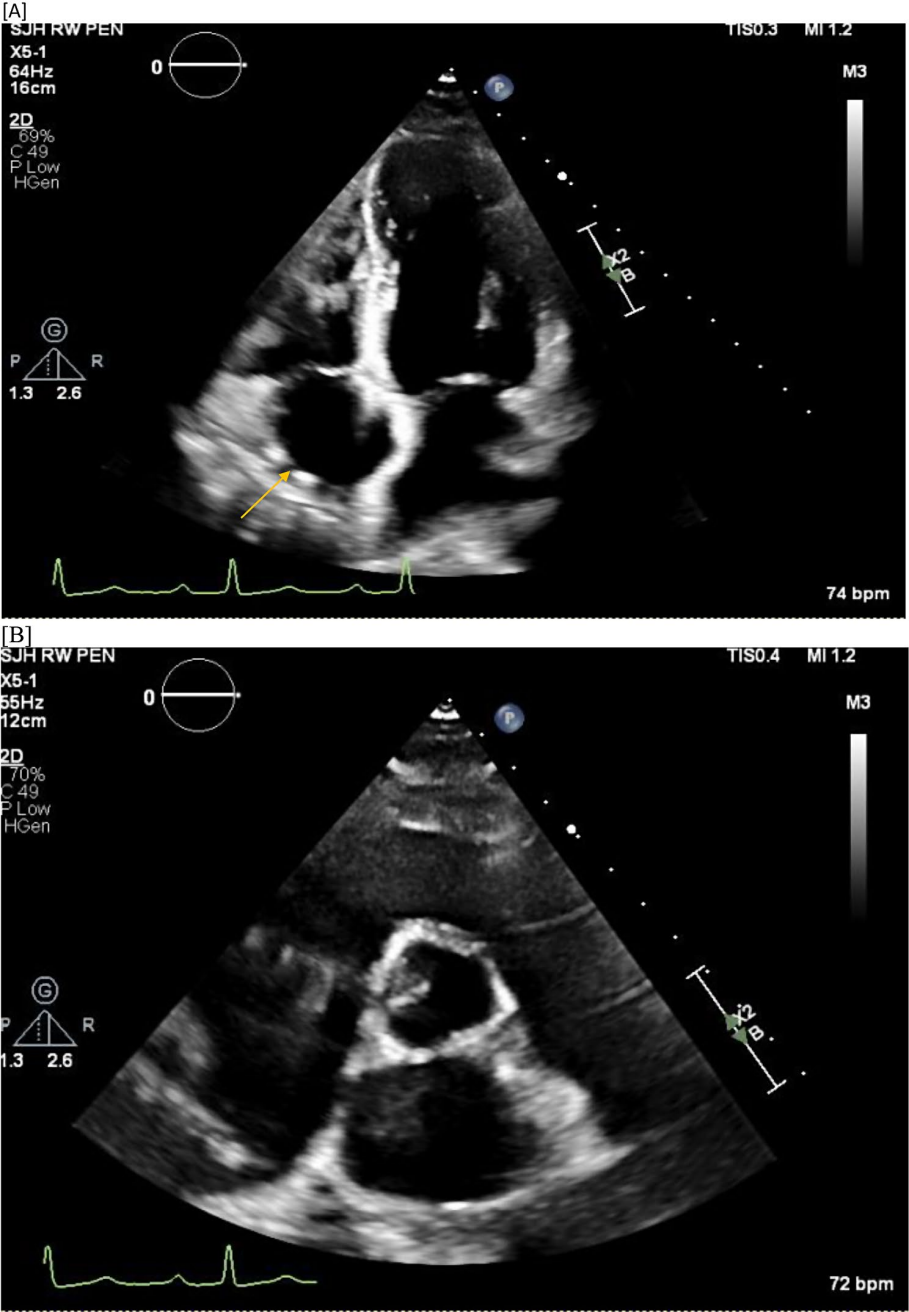
TTE Post surgical removal of intra-cardiac masses(**A**-**B**)

TTE done post cardiac mass excision showed there were still 2 small, but prominent echogenic structures in the free wall of the right atrium though much smaller compared to previous TTE (Fig. 6).

Multiple malignancies like intracardiac myxoma and lymphoma were ruled out by a negative biopsy, absence of intra-cranial mets and other relevant investigations. Other rheumatological conditions that could present as intra-cardiac masses like giant cell myocarditis and rheumatoid nodule were duly ruled out by the absence of relevant family history, clinical findings and negative rheumatological work up.

Cardiac amyloidosis too can present with delayed gadolinium enhancement but was ruled out in this patient.

Angiosarcoma was ruled out only after a complete surgical resection of the mass since the biopsy of the masses did reveal atypical spindle cells, which in hindsight were benign and thought to occur due to the associated inflammation caused by sarcoidosis. Following removal of intra-cardiac masses; patient underwent removal of temporary pacemaker and insertion of a dual-chamber ICD implant to treat the complete heart block that had resulted from the progressive conduction disease due to sarcoid; with current rhythm being ventricularly paced at 60-80 bpm.

Patient was started on 30 mg methylprednisone IV daily with eventual tapering, cholecalciferol 200-400 mg for the low vitamin D levels and on outpatient started on infliximab 5 mg/kg q8h and methotrexate 7.5 mg/week. Patient was scheduled for a regular follow up of 3 months after which a TTE would be done to check for disease progression/ clinical remission.

## Discussion

Heart block or progressive conduction pathway disease is one of the most common presenting manifestations in patients with cardiac sarcoidosis observed to have an abnormal electrocardiogram. This phenomenon may primarily result from granulomatous infiltration of the interventricular septum or nodal artery, leading to concomitant ischemia and disruption of the conduction system [5, 6].

The reason for conduction anomalies in our case can be explained by two schools of thoughts; one which focuses on presence of the mass demonstrated on ICE, which due to it’s close proximity AV node, which is situated in the posterior aspect of the interatrial septum near the orifice of the coronary sinus. The extension of the mass into this area could potentially involve or impinge upon the AV node, leading to the observed conduction abnormalities. Another possible explanation is the patchy nature of the disease that could involve multiple foci within the heart. These patchy areas of granulomatous inflammation, though not directly involving the primary masses, could potentially extend to critical regions like the basal interventricular septum and the AV node, leading to conduction disturbances. Therefore, even in the absence of direct mass effect on the AV node, the diffuse and multi-focal nature of sarcoid involvement could account for the progressive conduction system disease observed in this case [7].

Other presenting symptoms can be ventricular tachyrythmias, which are often a result of atrial granulomas, sudden cardiac death, congestive heart failure and rarely pericardial/valvular involvement. [8].

According to the Expert Consensus Recommendations on Criteria for Diagnosis of Cardiac Sarcoidosis by Birnie et al., our case meets the diagnostic criteria for cardiac sarcoidosis, as there is the presence of non-caseating granulomas on endomyocardial biopsy, extra-cardiac manifestation as hilar adenopathy, along with features such as heart block and patchy uptake on 18 F-FDG PET imaging. The latter also demonstrates that hilar/mediastinal lymph nodes can be metabolically active without being enlarged, as seen in this patient [9].

Focal on diffuse FDG distribution pattern likely indicates cardiac sarcoidosis, and can help point towards a diagnosis even when endomyocardial biopsy is negative(due to low sensitivity of < 25%) and late gadolinium enhancement on a cardiac morphology MRI is invariably diagnostic for CS; both of which were seen in the case above [10].

Ori et al. suggests that even though FDG-PET is more sensitive in detecting CS as compared to cardiac MRI since scarring may not always be present in CS but using both in the diagnostic approach towards CS increasing the probability of diagnosing it early and starting proper management. However for follow up in CS patients CMR does not have an important role since most CS patients have an implantable device(as seen in our patient) [11].

The treatment received by the patient is parallel to the general recommendations which involve corticosteroids being the primary pharmacological therapy to delay the disease’s natural course and stop fibrosis; at least over a short duration of time following which FDG-PET needs to be repeated (likely after 3 months) to check for disease progression and response. Non-pharmacological approach utilising implantable cardiac devices like pacemakers and defibrillators has improved the prognosis of CS, as scar formation promotes more VTs which can be controlled by ICRT-D/P [12].

While it is true that steroid treatment can be effective in some cases of cardiac sarcoidosis, the case clearly outlines the rapidly progressive nature of the patient’s conduction disease, culminating in complete heart block. Patel et al.demonstarted in patients with isolated cardiac sarcoidosis less profound effect of steroids in ameliorating the conduction system manifestations of the disease. This, coupled with the rapidly progressive nature of the patient’s conduction block, necessitated permanent ICD placement [13].

Certain international consensus guidelines recommend the use of methotrexate and tumor necrosis factor-alpha inhibitors like infliximab or adalimumab in severe or refractory cases of cardiac sarcoidosis, especially as second- and third-line agents, which also aim to minimize steroid exposure. It is also crucial to monitor certain biomarkers like neopterin or soluble interleukin-2 receptor alpha, which can be utilized to track disease activity and will be measured during follow-up in this patient [14].

Obi et al. assert that initiating immunosuppressive therapy earlier in the course of cardiac sarcoidosis yields better outcomes, with its primary indication being the presence of clinically relevant disease manifestations (as seen in this case). Methotrexate, with its anti-inflammatory action in conjunction with concurrent steroid therapy, has been demonstrated to improve survival and promote disease resolution in cardiac sarcoidosis patients. Additionally, emerging data on the use of infliximab in treating cardiac sarcoidosis, particularly in the presence of significant cardiac involvement, further substantiates the rationale for its administration to our patient [15].

It is noteworthy that there are only a handful of rare cases worldwide that discuss in detail how sarcoidosis can unusually present as an intra-cardiac tumor and how it needs to be differentiated from other potential intra-cardiac masses. Our patient is expected to make a full recovery, as was the case elaborated by Powell et al., which also demonstrated that using steroids as primary immunosuppressants facilitated disease resolution [16].

In cases of cardiac sarcoidosis with advanced and refractory disease, particularly when associated with severe left ventricular dysfunction or life-threatening ventricular arrhythmias refractory to medical therapy and catheter ablation, cardiac transplantation may be considered as a last resort. According to the International Society for Heart and Lung Transplantation guidelines, cardiac sarcoidosis is a recognized indication for heart transplantation in the setting of end-stage heart failure or incessant ventricular arrhythmias despite optimal medical management [17]. In the present case, although the patient had conduction system disease requiring an ICD, there was no evidence of advanced cardiac dysfunction or refractory arrhythmias to warrant consideration of transplantation at this stage. Nonetheless, close monitoring and timely escalation of therapy, including potential transplant evaluation, may be warranted if disease progression occurs despite optimal medical management.

## Conclusion

This case illustrates that cardiac sarcoidosis can manifest in atypical and deceptive ways, challenging the conventional paradigms of disease presentation.By meticulously documenting this unique presentation and highlighting the diagnostic and therapeutic nuances, this report contributes to the expanding understanding of cardiac sarcoidosis and its diverse clinical spectrum. It serves as a valuable addition to the limited body of literature on this rare and challenging condition, providing insights that may guide clinicians in recognizing and managing similar cases in the future.

Ultimately, this case underscores the importance of maintaining a high index of suspicion, employing a multimodal diagnostic approach, and tailoring treatment strategies to individual patient needs in the management of cardiac sarcoidosis.

## Data Availability

No datasets were generated or analysed during the current study.

## References

[1] Sreeja C, et al. Sarcoidosis - a review article. J Oral Maxillofac Pathol. 2022;26(2):242. www.ncbi.nlm.nih.gov/pmc/articles/PMC9364657/. 10.4103/jomfp.jomfp_373_21. 10.4103/jomfp.jomfp_373_21 PMC9364657 35968162

[2] Baughman Robert P, et al. Clinical characteristics of patients in a case control study of Sarcoidosis. Am J Respir Crit Care Med. 2001;164(10):1885–9. 10.1164/ajrccm.164.10.2104046. 10.1164/ajrccm.164.10.2104046 11734441

[3] Patel MR, et al. Detection of myocardial damage in patients with Sarcoidosis. Circulation 2009;120(20):1969–77. 10.1161/circulationaha.109.851352. Accessed 4 Oct 2022. 10.1161/CIRCULATIONAHA.109.851352 PMC2778859 19884472

[4] Lynch JP et al. Cardiac involvement in Sarcoidosis: evolving concepts in diagnosis and treatment. Semin Respir Crit Care Med. 2014;35(03):372–390. 10.1055/s-0034-1376889. Accessed 26 Apr 2023. 10.1055/s-0034-1376889 PMC4253029 25007089

[5] Fleming HA. Sarcoid Heart Disease. Heart. 1980;43(3):366–366. 10.1136/hrt.43.3.366. Accessed 27 Nov 2019. 10.1136/hrt.43.3.366 PMC482291 7437186

[6] Matsui Y, et al. Clinicopathological study on fatal myocardial sarcoidosis. 278(1 Seventh Inter):455–69. 10.1111/j.1749-6632.1976.tb47058.x. Accessed 23 May 2023. 10.1111/j.1749-6632.1976.tb47058.x 1067031

[7] Nery PB, Beanlands RS, Nair GM, Green M, Yang J, McArdle BA, et al. Atrioventricular block as the initial manifestation of cardiac sarcoidosis in middle-aged adults. J Cardiovasc Electrophys. 2014;25(8):875–81. 10.1111/jce.12401 24602015

[8] Roberts WC, McAllister HA Jr, Ferrans VJ. Sarcoidosis of the Heart. A clinicopathologic study of 35 necropsy patients (Group I) and review of 78 previously described necropsy patients (Group II). Am J Med. 1977;63(1):A81. 10.1016/0002-9343(77)90145-0. Accessed 13 Nov 2021. 10.1016/0002-9343(77)90121-8 327806

[9] Birnie DH et al. HRS expert consensus statement on the diagnosis and management of arrhythmias associated with cardiac sarcoidosis. Heart Rhythm. 2014;11(7):1304–23. 10.1016/j.hrthm.2014.03.043. Accessed 2 June 2020. 10.1016/j.hrthm.2014.03.043 24819193

[10] Ishimaru S et al. Focal uptake on 18F-Fluoro-2-Deoxyglucose Positron emission tomography images indicates cardiac involvement of sarcoidosis†. Eur Heart J. 2005;26(15):1538–43. 10.1093/eurheartj/ehi180. Accessed 4 Dec 2022. 10.1093/eurheartj/ehi180 15809286

[11] Orii M, Imanishi T, Akasaka T. Assessment of cardiac sarcoidosis with advanced imaging modalities. Biomed Res Int. 2014;2014:1–15. 10.1155/2014/897956 PMC4163361 25250336

[12] Kusano KF, Satomi K. Diagnosis and treatment of cardiac sarcoidosis. Heart 2016;102(3):184–90. heart.bmj.com/content/102/3/184. 10.1136/heartjnl-2015-307877. 10.1136/heartjnl-2015-307877 26643814

[13] Petersen MR, Perry C, Nickels R. Isolated cardiac sarcoidosis with high-grade heart block: utilization of new diagnostic guidelines. In: Ercan E, editor. Case reports in cardiology. 2021;2021:1–5. 10.1155/2021/9992678 PMC8342175 34367698

[14] Prasse A. The diagnosis, differential diagnosis, and treatment of sarcoidosis. Deutsches Aerzteblatt Online. 2016. 10.3238/arztebl.2016.0565. 10.3238/arztebl.2016.0565 PMC5015588 27598883

[15] Obi ON, Lower EE, Baughman RP. Controversies in the treatment of cardiac sarcoidosis. 2022;39(2):e2022015–5. https://www.ncbi.nlm.nih.gov/pmc/articles/PMC9437759/. [cited 2023 Jul 6]. 10.36141/svdld.v39i2.13136 PMC9437759 36118546

[16] Powell RGG, et al. Multiple intramyocardial masses in an otherwise healthy 35-year-old woman. CJC Open 2022;4(4):432–4. 10.1016/j.cjco.2021.12.009. Accessed 26 Feb 2023. 10.1016/j.cjco.2021.12.009 PMC9039576 35495854

[17] Mehra MR, Canter CE, Hannan MM, et al. The 2016 International Society for Heart Lung Transplantation listing criteria for heart transplantation: a 10-year update. J Heart Lung Transpl. 2016;35(1):1–23. 10.1016/j.healun.2015.10.023 26776864

